# Chronically Retained Central Venous Catheter in Deceased Donor Liver Allograft

**DOI:** 10.1155/2019/4359197

**Published:** 2019-04-30

**Authors:** Shannon Zielsdorf, Beau Kelly, Yuri Genyk, Juliet Emamaullee

**Affiliations:** ^1^Department of Surgery, University of Southern California, Los Angeles, CA, USA; ^2^Sierra Donor Services, Sacramento, CA, USA

## Abstract

Central venous catheters (CVC) are commonly used across multiple medical specialties and are inserted for various reasons. A known, but rare, serious complication of CVC is fracture and retention of residual catheter. Here we describe a chronically retained catheter within the inferior vena cava (IVC) that was asymptomatic and neither diagnosed nor addressed until time of deceased donor liver donation. Prior to transplantation into the recipient, the retained catheter was removed, and a venoplasty of the suprahepatic IVC, middle hepatic vein, and left hepatic vein was performed with no significant issues after transplant in the recipient. With the persistent shortage of suitable organs for transplant leading to patients dying on the waiting list, every good quality organ should be carefully considered. Thus, even though a chronically retained, fractured CVC in a deceased organ donor presents a unique challenge, it can be managed surgically and should not be considered a contraindication to organ utilization.

## 1. Introduction

Central venous catheters (CVC) are commonly used across multiple medical specialties and are inserted for various reasons, such as administration of long-term antibiotics, parenteral nutrition, and chemotherapy. A serious, but rare, complication of CVC is fracture and retention of residual catheter, in up to 2.2% of all line insertions [1]. There are multiple case reports describing catheter rupture and migration to the right ventricle (reviewed in [2]). Typically, treatment involves endovascular retrieval of the retained foreign object, if possible [3]. In this case report, we describe for the first time a chronically retained CVC within the inferior vena cava (IVC) that was neither diagnosed nor addressed until time of deceased donor liver donation.

## 2. Case Report

**Figure 1:**
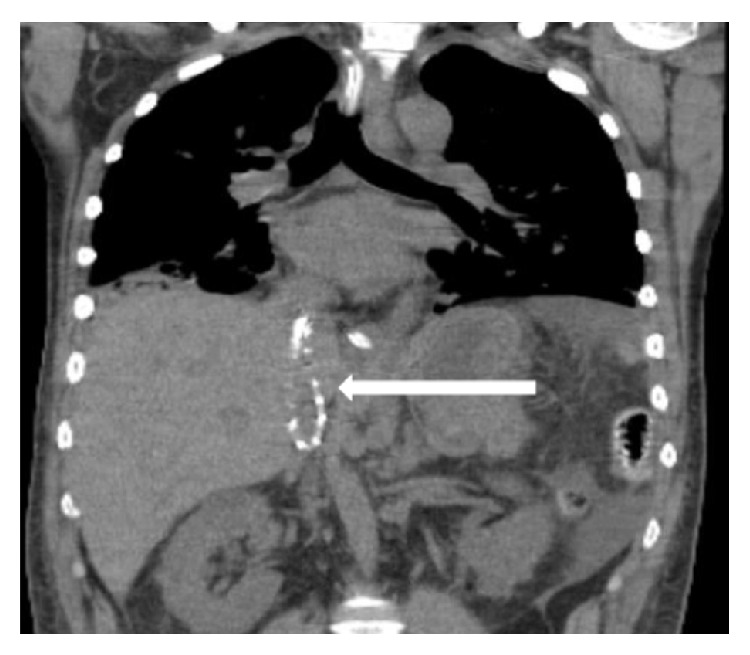
*Evidence of retained CVC on predonation CT scan.* White arrow pointing at retained CVC in IVC (coronal view).

A 46-year-old male with a history of severe developmental delay, hydrocephalus, and seizure disorder presented to the hospital with blunt head trauma after a ground level fall. Work-up revealed bilateral acute subdural hematomas for which an external ventricular drain was placed. Despite intensive care management, the patient deteriorated to brain death. He was subsequently evaluated for organ donation. Abdominal computerized tomography (CT) scan (Figure 1) revealed an “elongated structure with metallic components in the upper portion of the IVC that extends into the right atrium.” There was no medical history of a prior procedure, or symptoms, to explain the incidental finding. The radiologist's interpretation and presumptive diagnosis were a retained atrial pacing wire.

**Figure 2:**
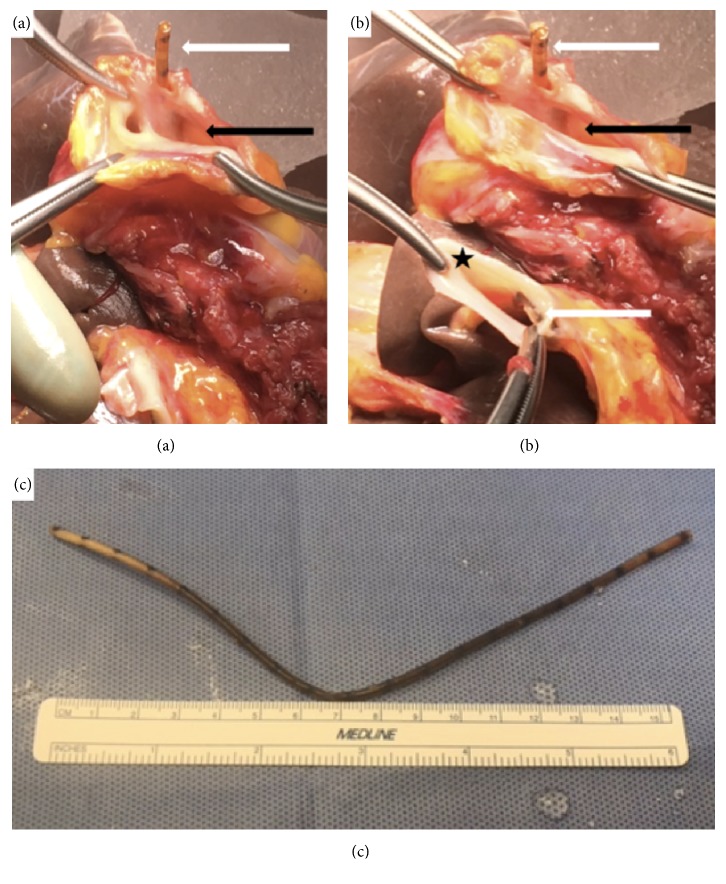
*Retained CVC in donor liver, postprocurement.* Panel (a): suprahepatic IVC. White arrow pointing at retained CVC within IVC wall. Black arrow pointing at orifice of MHV and LHV. Panel (b): white arrows pointing at retained CVC within IVC wall. Black arrow pointing at orifice of MHV and LHV. Black star at infrahepatic IVC. Panel (c): retained CVC after removal.

**Figure 3:**
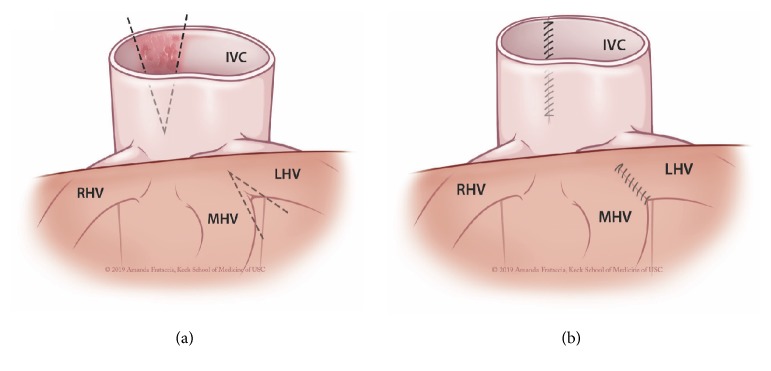
*Method of venoplasty for removal of CVC cast in donor IVC prior to transplantation.* Panel (a): anterior view of the native donor suprahepatic caval anatomy prior to venoplasty, with an area of inflammation and calcification due to foreign body reaction. Panel (b): anterior view postvenoplasty, demonstrating the areas of the IVC and MHV/LHV junction that were repaired to improve venous outflow and the overall integrity of the suprahepatic caval anastomosis.

He subsequently underwent procurement for organ donation after brain death. At the time of cross-clamp, the previously identified foreign body was transected when the right atrium was incised for exsanguination. During the back-table dissection, it was apparent that the foreign body had eroded into the posterior wall of the IVC, extending down the retrohepatic IVC (Figures 2(a) and 2(b)). It also created a calcified reaction at the junction of the suprahepatic IVC and right atrium, adjacent to the left hepatic vein (LHV) and middle hepatic vein (MHV). We removed the foreign body (Figure 2(c)) and performed a venoplasty (Figure 3) of the posterior wall of the IVC and of the common wall of the LHV and MVH, so that the outflow of the LHV and MHV was not compromised after transplant. We discovered that the foreign body was, most likely, a fractured CVC due to the overall appearance and interval markings.

**Figure 4:**
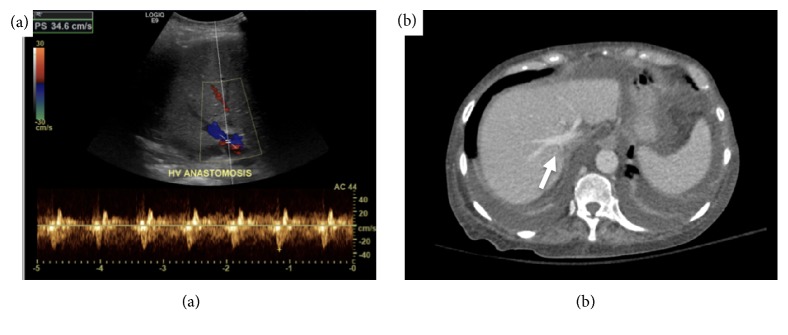
*Posttransplant imaging reveals no sequelae of removal of retained CVC.* Panel (a): liver ultrasound with Doppler showing normal hepatic venous anastomosis waveforms and velocities. Panel (b): axial contrasted CT scan showing patent, normal appearing hepatic venous anastomosis (arrow).

The liver recipient was a 65-year-old woman with cirrhosis due to alcohol abuse; her Na-MELD score was 40 at the time of transplant. She underwent caval-sparing total hepatectomy and deceased donor liver transplantation via piggyback technique: the donor suprahepatic IVC was anastomosed to a common orifice of the recipient's right and middle hepatic veins. We did not alter our immunosuppressive therapy or prophylactic antibiotic regimen. Additionally, we did not initiate any anticoagulants or antiplatelet agents beyond our standard postoperative protocol. Postoperative imaging showed normal velocities and waveforms on ultrasound (Figure 4(a)) and unremarkable appearance on axial CT (Figure 4(b)) of the hepatic vein anastomosis. The patient otherwise had an uneventful postoperative course and has had stable allograft function with no venous outflow issues for >8 months after transplant. There were no reported complications in the other organ recipients.

## 3. Discussion

Retained CVC is a rare complication that often results in fractured components that migrate to the right heart. The mainstay treatment, regardless of symptoms, is urgent endovascular removal [3]. In the present case report, we describe for the first time a patient with a chronically retained CVC that did not migrate and instead became encased within the posterior wall of the IVC. Furthermore, the CVC erosion into the intima caused an inflammatory response that created a calcified “cast” within the vessel wall. The mechanism behind CVC retention is likely linked to this calcified “cast.” According to Aworanti et al., the typical fibrin sheath that develops around a catheter can undergo epithelialization, calcification, and then adhere to the tunica intima of the vein wall [1]. While vascular calcifications typically occur in arteries, a central vein calcification is rare but has been described [4]. Despite being retained for over six years with previous failed attempts at catheter removal, Capitanini described the development of a calcified “cast” within the superior vena cava, seen on CT scan approximately eight months after extraction. No additional treatment was offered because the patient remained asymptomatic [4]. This is consistent with the findings of Aworanti et al. that suggested that chronic use of CVC is a common factor when retained catheters are encountered [1]. In this study, out of 174 pediatric CVC insertions and 135 subsequent removals, three patients (2.2%) failed removal; all three patients had had their CVC for greater than six years.

In the present case, it is presumed that the CVC was in place for a prolonged period of time given the patient's history of recurrent infections as well as chronic disability requiring residency in a long-term care facility. In addition, unlike the case report by Capitanini, the calcified wall of the IVC warranted intervention for two reasons: first, it was located at the MHV/LHV junction with the IVC and second, this area was to be anastomosed in the transplant recipient. A venoplasty was necessary to prevent any impediment of venous outflow from the allograft and to ensure a secure anastomosis, respectively.

In conclusion, the shortage of donor livers for transplant necessitates careful evaluation of every suitable organ as a potential life-saving treatment option for the appropriate waitlisted candidate. A chronically retained, fractured CVC in a deceased organ donor, with an otherwise normal liver, presents a unique challenge that can be successfully managed surgically. While care should be taken to ensure that there is neither impediment to hepatic venous outflow nor any nidus for future infection or thrombus formation, a chronically retained CVC should not be considered a contraindication to liver allograft utilization.

## Abbreviations

CVC: Central venous catheter
IVC: Inferior vena cava
CT: Computerized tomography
LHV: Left hepatic vein
MHV: Middle hepatic vein.
